# Super-enhancer receives signals from the extracellular matrix to induce PD-L1-mediated immune evasion *via* integrin/BRAF/TAK1/ERK/ETV4 signaling

**DOI:** 10.20892/j.issn.2095-3941.2021.0137

**Published:** 2021-10-09

**Authors:** Panpan Ma, Xinxin Jin, Zhiwei Fan, Zhou Wang, Suhui Yue, Changyue Wu, Shiyin Chen, Yuanyuan Wu, Miaomiao Chen, Donghua Gu, Siliang Zhang, Renfang Mao, Yihui Fan

**Affiliations:** 1 Laboratory of Medical Science, School of Medicine, Nantong University, Nantong 226001, China; 2 Department of Pathogenic Biology, School of Medicine, Nantong University, Nantong 226001, China; 3 Department of Clinical Laboratory, Yancheng No. 1 People’s Hospital, Yancheng 224005, China; 4 School of Life Sciences, Nantong University, Nantong 226001, China; 5 Department of Dermatology, Affiliated Hospital of Nantong University, Nantong University, Nantong 226001, China; 6 The Department of Urology, the Second Affiliated Hospital of Nantong University, Nantong University, Nantong 226001, China; 7 The Department of Radiotherapy Oncology, Harbin Medical University Cancer Hospital, Harbin 150086, China; 8 Department of Pathophysiology, School of Medicine, Nantong University, Nantong 226001, China

**Keywords:** PD-L1, super-enhancer, ETV4, ERK, αvβ3 integrin, BRAF, TAK1

## Abstract

**Objective:**

PD-L1 and PD-L2 expression levels determine immune evasion and the therapeutic efficacy of immune checkpoint blockade. The factors that drive inducible PD-L1 expression have been extensively studied, but mechanisms that result in constitutive PD-L1 expression in cancer cells are largely unknown.

**Methods:**

DNA elements were deleted in cells by CRISPR/Cas9-mediated knockout. Protein function was inhibited by chemical inhibitors. Protein levels were examined by Western blot, mRNA levels were examined by real-time RT-PCR, and surface protein expression was determined by cellular immunofluorescence and flow cytometry. Immune evasion was examined by *in vitro* T cell-mediated killing.

**Results:**

We determined the core regions (chr9: 5, 496, 378–5, 499, 663) of a previously identified PD-L1L2-super-enhancer (SE). Through systematic analysis, we found that the E26 transformation-specific (ETS) variant transcription factor (ETV4) bound to this core DNA region but not to DNA surrounding PD-L1L2SE. Genetic knockout of ETV4 dramatically reduced the expressions of both PD-L1 and PD-L2. ETV4 transcription was dependent on ERK activation, and BRAF/TAK1-induced ERK activation was dependent on extracellular signaling from αvβ3 integrin, which profoundly affected ETV4 transcription and PD-L1/L2 expression. Genetic silencing or pharmacological inhibition of components of the PD-L1L2-SE-associated pathway rendered cancer cells susceptible to T cell-mediated killing.

**Conclusions:**

We identified a pathway originating from the extracellular matrix that signaled *via* integrin/BRAF/TAK1/ERK/ETV4 to PD-L1L2-SE to induce PD-L1-mediated immune evasion. These results provided new insights into PD-L1L2-SE activation and pathways associated with immune checkpoint regulation in cancer.

## Introduction

Cancer immunotherapy can be broadly divided into 4 categories, including immune checkpoint inhibitors (ICIs), chimeric antigen receptor T-cell therapy, cytokines, and vaccines. Unlike conventional therapies such as surgery, chemotherapy, and radiotherapy, cancer immunotherapy activates the body’s immune system to fight cancer cells, and has become an innovative tool used to treat more than a dozen types of cancers^1–3^. Immune checkpoints are essential for maintaining immune homeostasis and preventing autoimmunity; nevertheless, they are often activated to suppress nascent antitumor immune responses^4,5^. Among the identified immune checkpoints, cytotoxic T-lymphocyte antigen-4 (CTLA-4) and programmed cell death protein 1 (PD-1) are 2 receptors that have shown promising therapeutic outcomes^6^. To escape immune cell-mediated killing, a variety of cancer cells present the PD-1 ligand 1 (PD-L1) on their surface to restrict full activation of T cells they encounter^7^. Although anti-PD-1/PD-L1 drugs provide a new breakthrough treatment with promising longer-term efficacy, their benefits to the overall population of cancer patients are quite low due to low responses and immune-related adverse events^8,9^. Thus, identifying the underlying systems that are activated by cancer cells to achieve expression of inhibitory checkpoint molecules will provide new insights that will improve responses and reduce side effects.

PD-L1, also known as cluster of differentiation 274 (*CD274*) or B7 homolog 1 (B7-H1), is encoded by the *CD274* gene. Programmed cell death 1 ligand 2 (PD-L2), which is encoded by *CD273* is homologous to PD-L1 and shows higher native binding affinity to PD-1, when compared with PD-L1^10,11^. In normal healthy conditions, PD-L1 and PD-L2 are primarily expressed on antigen-presenting cells and activated immune cells to turn-off immune responses and re-establish immune homeostasis after activation^12^. However, during oncogenesis, cancer cells organize different programs to activate PD-L1 and PD-L2 expressions^13^. For example, a multitude of cytokines in the tumor microenvironment such as IFNγ, TNFα, and IL-6 can initiate or increase PD-L1 expression^14,15^. Moreover, oncogenic pathways such as RAS, MYC, and STAT3 signaling can induce constitutive PD-L1 expression^14,15^. PD-L1 and PD-L2 expressions in cancer determine responses to ICIs, and also affect the establishment of intrinsic and acquired resistance to ICIs. Thus, fully understanding the molecular mechanisms of activation by cancer cells to induce PD-L1 and PD-L2 expressions is important in the development of accurate and individualized ICI treatments. Recently, we identified a super-enhancer (SE) named PD-L1L2-SE, which was essential for constitutive PD-L1 and PD-L2 expressions^16^. Deleting PD-L1L2-SE in cancer cells is sufficient to robustly reduce PD-L1 and PD-L2 expressions, which subsequently makes the cells sensitive to T cell-mediated killing^16^. However, the extracellular signals and critical transcription factors (TFs) that activate PD-L1L2-SE in cancer cells remain to be determined.

The ETS-TFs have a conserved ETS domain that recognizes a purine-rich core DNA sequence^17,18^. There are 28 members of the ETS family in the human genome, with most being involved in crucial biological processes such as development, differentiation, cell death, and angiogenesis. Furthermore, many ETS TFs are implicated in cancer initiation and progression. One ETS subfamily named polyomavirus enhancer activator 3 (PEA3) includes 3 homologous members: ETV1, ETV4, and ETV5^19^. Among these, ETV4 has been widely reported to play oncogenic roles in numerous cancers such as papillary thyroid carcinoma, renal cell carcinoma, gastric cancer, lung cancer, and breast cancer^20^. However, the molecular mechanism through which ETV4 promotes carcinogenesis remains largely unknown. It is also unclear whether ETV4 is involved in immune evasion and SE activation. Here, by precisely mapping the identified PD-L1L2-SE, we discovered that ETV4 was a critical TF that induced PD-L1L2-SE-mediated PD-L1 and PD-L2 expressions. ETV4 was transcriptionally activated by αvβ3 integrin through the TAK1/BRAF/ERK pathway. Importantly, cancer cells with genetic knockout or pharmacological inhibition of components of this pathway were sensitive to T cell-mediated killing.

## Materials and methods

### Cell culture and treatment

MCF7, MDA-MB-231, and SUM-159 cells were cultured in Dulbecco’s Modified Eagle’s Medium (Hyclone, Logan, UT, USA) supplemented with 10% fetal bovine serum (FBS), 100 U/mL penicillin, and 100 mg/mL streptomycin. All cell lines were cultured in a humidified atmosphere of 5% CO_2_ at 37 °C. For treatments with the following inhibitors, cells were seeded at the same density and allowed to adhere for 24 h under normal conditions before treatment with the indicated concentrations of different inhibitors. The inhibitors including AZD8330 (A8374), SB203580 (A8254), SP600125 (A4604), PS-1145 (B6089), (5Z)-7-Oxozeaenol (B7443), MK2206 (A3010), LY2109761 (A8464), and Cyclo-RGDfK (A8164) were purchased from APExBIO (Houston, TX, USA). Belvarafenib (S8853) was purchased from Selleck Chemicals (Houston, TX, USA), and UK122 (sc-356185) was purchased from Santa Cruz Biotechnology (Santa Cruz, CA, USA).

### RNA isolation and quantitative real-time PCR

Total RNA was extracted from cells treated with various inhibitors using the traditional TRIzol method (Invitrogen, Carlsbad, CA, USA). The purity and concentration of total RNA were evaluated using a Nanodrop 1000 spectrophotometer (Thermo Fisher Scientific, Waltham, MA USA). Isolated RNA was then reverse transcribed to cDNA according to the instructions of the HiScript^®^ II QRT SuperMix for qPCR (+gDNA wiper) kit (R223-01; Vazyme, Nanjing, China). Finally, relative expressions of the indicated genes were detected using the AceQ^®^ qPCR SYBR Green Master Mix kit (Q111-02; Vazyme). The primers used are listed below: human PD-L1 (forward: 5′-GTAGCACTGACAT TCATCTTC-3′, reverse: 5′-TTCCTTCCTCTTGTCACGCTC-3′); human PD-L2 (forward: 5′CATAGCCACAGTGATAGCCCT-3′, reverse: 5′-GGCTCCCAAGACCACAGGTTC-3′); human ETV4 (forward: 5′-CAGTGCCTTTACTCCAGTGCC-3′, reverse: 5′-CT CAGGAAATTCCGTTGCTCT-3′); human RNF2 (forward: 5′-GT GCCATACTAAGCAGCTTGC-3′, reverse: 5′-ACACTACAGGTC GGAAATCCA-3′); human BRD3 (forward: 5′-ATCACTGCA AACGTCACGTC-3′, reverse: 5′-CCTGCTTGGGGTCTGACA AC-3′); human IFNγ (forward: 5′-GGTTCTCTTGGCTGTTAC TG-3′, reverse: 5′-ATCCGCTACATCTGAATGAC-3′); and human granzyme B (forward: 5′-GACAGTACCATTGAGTTGTGC-3′, reverse: 5′-CTGGGCCACCTTGTTACACAC-3′).

### Western blot assays

Cells were lysed in radioimmunoprecipitation assay buffer (WB3110; New Cell & Molecular Biotech, China) supplemented with protease and phosphatase inhibitors (P002; New Cell & Molecular Biotech, China). Protein concentrations were quantified using a BCA assay kit (WB6501; New Cell & Molecular Biotech, China), and the assays were read on a Beckman Coulter DU-800 (M200) spectrophotometer (Beckman, Brea, CA, USA). Equal amounts of protein were subjected to SDS-PAGE, and then transferred onto polyvinylidene fluoride membranes (GE Healthcare, Chicago, IL, USA). After blocking in 5% nonfat milk in TBST for 2 h at room temperature, the membranes were incubated with the following primary antibodies overnight at 4 °C: anti-PD-L1 (ab213524; Abcam, Cambridge, UK), anti-glyceraldehyde 3-phosphate dehydrogenase (GAPDH) (sc-365062; Santa Cruz Biotechnology), anti-HSP70 (sc-32239; Santa Cruz Biotechnology), and anti-HSP90 (sc-13119; Santa Cruz Biotechnology). After incubation with horseradish peroxidase-conjugated secondary antibodies for 2 h at room temperature, the blots were visualized using an ECL kit (BL520B; Biosharp, Sakai, Japan). Finally, the images were analyzed using ImageJ software (National Institutes of Health, Bethesda, MD, USA).

### Flow cytometry

Cells were seeded into a 6-well plate, and then treated with inhibitors for 48 h. The treated cells were collected and washed for 30 min in fluorescence-activated cell sorting (FACS) buffer [0.5% bovine serum albumin (BSA) and 2% FBS in phosphate-buffered saline (PBS)]. Phycoerythrin (PE)-conjugated anti-PD-L1 antibody and allophycocyanin (APC)-conjugated anti-PD-L2 antibody were added to the suspended cells for 30 min at 4 °C. After incubation, the cells were washed with FACS buffer (0.5% BSA in PBS) 3 times and detected using a BD Calibur (BD Biosciences, Franklin Lakes, NJ, USA) flow cytometer.

### Immunofluorescence staining

When the cell density reached 60% confluence on a round glass slide in a 24-well plate, the indicated inhibitors were added for 48 h. The samples were fixed with 4% paraformaldehyde for 15 min at room temperature, treated with cell permeabilization buffer (PBS with 0.2% Triton X-100) for 10 min at room temperature, and then blocked with blocking buffer (PBS with 1% BSA) for 2 h at room temperature. Primary antibodies were incubated with the cells overnight at 4 °C in the blocking solution. After washing in PBS with 0.25% FBS, secondary antibodies including anti-rabbit Alexa Fluor 488 or 594 dye conjugate (Jackson Laboratories, Bar Harbor, ME, USA) were added and incubated for 2 h at room temperature. Finally, the cells were incubated with Hoechst dye (33342; Beyotime, Nanjing, China) in the dark at room temperature for 10 min. To measure PD-1 and PD-L1 protein interactions, the indicated cells were fixed in 4% paraformaldehyde at room temperature for 30 min and then incubated with recombinant human PD-1-Fc protein (PKSH033554; Elabscience, Houston, TX, USA) for 2 h. The secondary antibody was anti-human Fc conjugated with PE (ab99761; Abcam). Fluorescence signals were detected and captured using a fluorescent microscope (DMI3000B; Leica, Wetzlar, Germany).

### Establishment of stable genetically-modified cell lines

The targeting oligonucleotides (sgC1-F: 5′-GTTGTAAACTGAG CATGCAA-3′, sgC1-R:5′-GTGACAATGCAATGTTGAAG-3′, sg C2-R:5′-GATCTTCCCAGATTTTCAGA-3′, sgC3-R:5′-GACT CCCATACAACAATAGG-3′, and sgETV4:5′-GCTGGGGAAG CTCATGGACC-3′) were cloned into the epiCRISPR vector. To establish stable genetic knockout cell lines, SUM-159 cells were co-transfected with a combination of designed sgRNAs. After 48 h of transfection, the medium was replaced with complete medium containing 2 μg/mL puromycin. Puromycin was used in the cell culture medium for an additional 2 weeks. The puromycin resistant cells were lysed with 50 μL of buffer L with 1 μL of protease for 30 min at 52 °C. The lysate was then used for genomic PCR and analyzed by agarose gel electrophoresis. DNA of the predicted size was purified for sequencing.

### Peripheral blood mononuclear cell (PBMC)-mediated killing in vitro

Human PBMCs were isolated from whole blood by density gradient centrifugation using a Ficoll-Paque buffer. Isolated PBMCs were cultured in RPMI-1640 supplemented with 10% FBS and were activated by a combination of anti-CD3 antibody, anti-CD28 antibody, and IL-2. The expanded T cells were collected for killing assays. Alternatively, CD8^+^ T cells were positively isolated by anti-CD8-FITC microbeads from PBMCs. SUM-159 cells and their genetically modified clones were seeded into 96-well plates. SUM-159 cells were also treated with inhibitors for 24 h as indicated. Activated and expanded T cells were counted and co-cultured with the SUM-159 cells at a 5:1 ratio. After 48 h, the T cells were washed with PBS and collected by centrifugation for RT-PCR. The remaining live cancer cells were photographed and quantified using the CCK-8 assay (Sigma-Aldrich, St. Louis, MO, USA). 

The ethics committees of Nantong University approved the study protocol (Approval Number: 2019-45), and the healthy donors provided written informed consent.

### Statistical analysis

Two-tailed Student’s *t*-test was used to determine the statistical significance between control and knockout cells. The statistical significance between groups treated with or without inhibitors was evaluated by a two-tailed Student’s *t*-test. All values are presented as the mean ± standard deviation (SD). A value of *P* < 0.05 was considered statistically significant. *P < 0.05; **P < 0.01; ***P < 0.001.

## Results

### Mapping the core DNA regulatory element in PD-L1L2-SE

**Figure 1:**
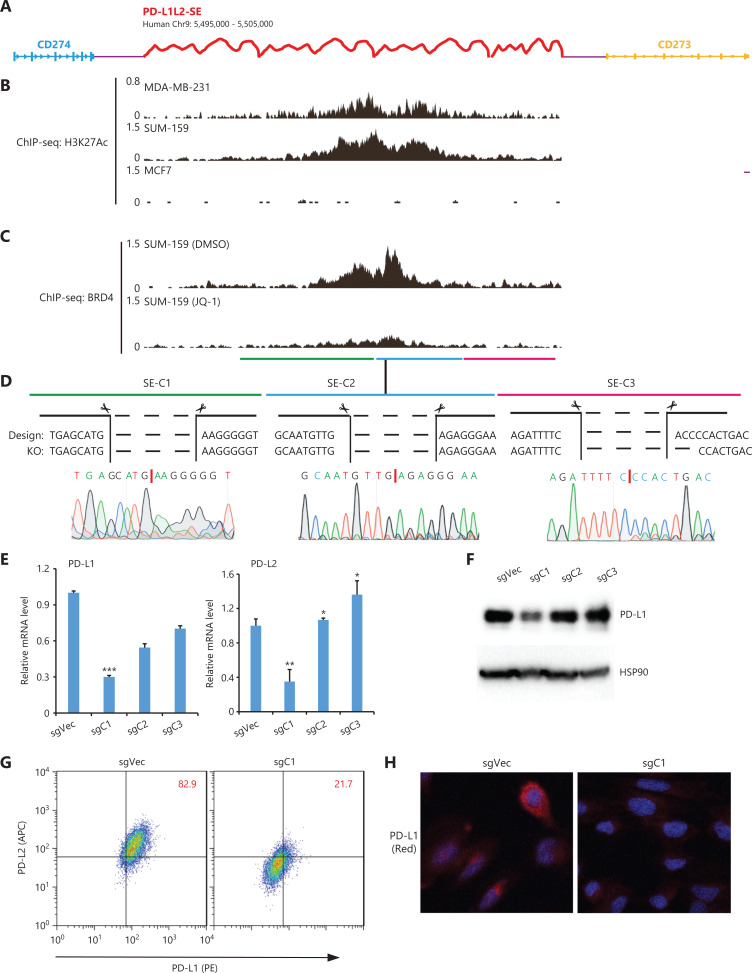
Functional analysis of DNA elements in PD-L1L2-SE. (A) Schematic representation of the genomic locations of *CD274*, *CD273*, and PD-L1L2-SE. (B) The distribution of H3K27Ac within Chr9: 5,495,000–5,505,000 in MCF-7, SUM-159, and MDA-MB-231 cells. The ChIP-seq data was downloaded from published datasets (GSE87424 and GSE85158). (C) BRD4 binding within Chr9: 5,495,000–5,505,000 before and after JQ-1 treatment. The ChIP-seq data were downloaded from a published dataset (GSM2330549). (D) The joint DNA sequences were determined by genomic PCR and sequencing. The PD-L1L2-SE was subdivided into 3 elements (C1, C2, and C3), which were individually deleted using CRISPR-Cas9 methodology. (E) Real-time PCR was used to determine the mRNA levels of PD-L1 and PD-L2 in DNA element-deficient (sgC1, sgC2, and sgC3) and control cells. (F) PD-L1 protein levels in control and DNA element-deficient cells were examined by Western blot. Hsp90 was used as the loading control. (G) The surface expressions of PD-L1 (PE-tagged) and PD-L2 (APC-tagged) in C1-deficient and control cells were analyzed by fluorescence-activated cell sorting. (H) Immunofluorescence was used to determine the expression and distribution of PD-L1 (red) in control and C1-deficient cells. Nuclei were stained with 4′,6-diamidino-2-phenylindole. *P < 0.05; **P < 0.01; ***P < 0.001.

In our previous study, we identified a SE (chr9: 5,495,000–5,505,000, 10 kb) called PD-L1L2-SE that was essential for constitutive PD-L1 and PD-L2 expression and PD-L1-mediated immune evasion in cancer. To further characterize the DNA regulatory element in PD-L1L2-SE, we divided it into 3 subdomains (C1, C2, and C3) based on enrichment of the H3K27Ac modification and BRD4 binding (**Figure 1A, 1B, and 1C**). Establishment of genetic knockouts for each element in SUM-159 cells was confirmed by PCR-based genotyping and sequencing (**Figure 1D** and data not shown). The joint DNA sequences after deleting each DNA element are shown in **Figure 1D**. We then assessed PD-L1 and PD-L2 expressions in C1-, C2-, and C3-deficient cells. RT-PCR showed that mRNA levels of both PD-L1 and PD-L2 were significantly reduced in cells with the C1 domain deletion (chr9: 5,496,378–5,499,663), but not in cells lacking the C2 or C3 elements (**Figure 1E**). C1-deficient cells also had greatly reduced PD-L1 protein levels compared with control cells (**Figure 1F**). The reduced PD-L1 protein expression in C1-deficient cells was confirmed by flow cytometry (**Figure 1G**). Consistently, immunofluorescence also demonstrated reduced PD-L1 expression in C1-deficient cells (**Figure 1H**). Together, these data showed that the C1 element (chr9: 5,496,378–5,499,663, ˜3 kb) was a core region of PD-L1L2-SE that was essential for PD-L1 and PD-L2 expressions.

### ETV4 drives PD-L1L2-SE-mediated PD-L1/L2 expression

**Figure 2:**
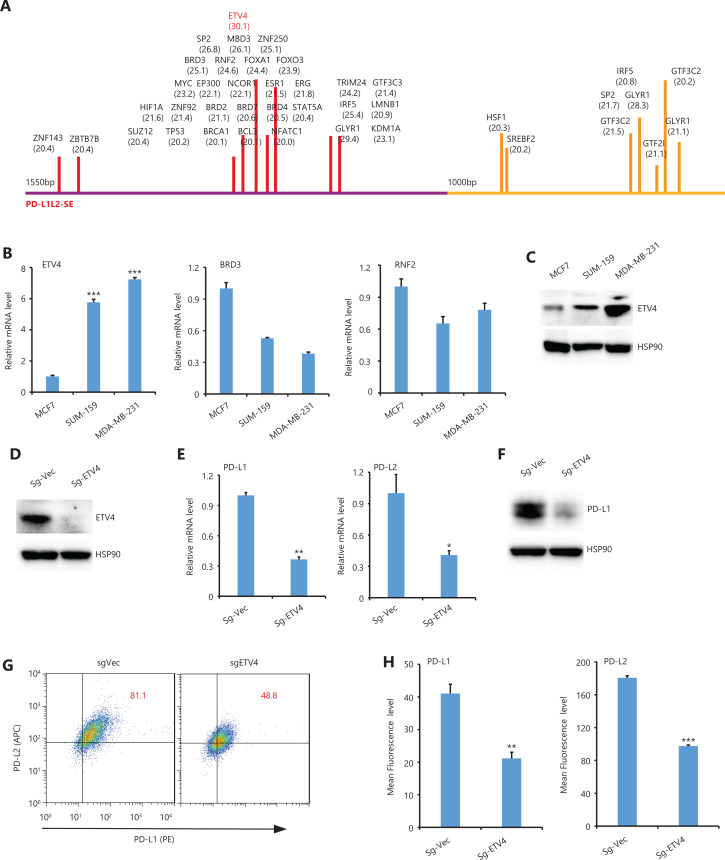
Analysis of the C1 and C2 elements and verification of ETV4 binding. (A) All transcription factors that potentially bind to the C1 and C2 BRD4-enriched regions are shown with their prediction scores. The analysis was performed using online programs: http://bioinfo.life.hust.edu.cn/AnimalTFDB/#!/predict (B) The relative mRNA levels of ETV4, BRD3, and RNF2 were determined by real-time RT-PCR in MCF7, SUM-159, and MDA-MB-231 cells. (C) ETV4 protein expressions in MCF7, MDA-MB-231, and SUM-159 cells were examined by Western blot. Hsp90 was used as the loading control. (D) ETV4 protein levels in control and ETV4-deficient (sgEtv4) cells were examined by Western blot. Hsp90 was used as the loading control. (E) Real-time PCR was used to determine the mRNA levels of PD-L1 and PD-L2 in control and ETV4-deficient cells. (F) PD-L1 protein levels in control and ETV4-deficient cells were examined by Western blot. Hsp90 was used as the loading control. (G) The surface expression of PD-L1 (PE) and PD-L2 (APC) in ETV4-deficient and control cells was analyzed by flow cytometry. (H) Summary of the mean fluorescence intensity from G. *P < 0.05; **P < 0.01; ***P < 0.001.

To investigate TFs that were important for PD-L1L1-SE-mediated PD-L1 expression, we systematically analyzed TFs that bound to the BRD4-enriched region of the C1 and C2 elements. Among all potential TFs that could bind, ETV4 had the highest score among all C1 bound TFs, while ETV4 did not bind the C2 element (**Figure 2A**). These results suggested that ETV4 might be important for PD-L1L2-SE-mediated PD-L1 expression. Because our previous study showed that PD-L1L2-SE was specifically activated in SUM-159 and MDA-MB-231 cells, but not MCF7 cells^16^, we then examined expression levels of 3 candidate TFs including ETV4, BRD3, and RNF2 in MCF7, SUM159, and MDA-MB-231 cells. Compared with MCF7 cells, ETV4 expression was significantly higher in both SUM-159 and MDA-MB-231 cells (**Figure 2B**). However, expressions of BRD3 and RNF2 in SUM-159 and MDA-MB-231 cells were relatively lower than in MCF7 cells (**Figure 2B**). The increased ETV4 expressions in SUM-159 and MDA-MB-231 cells were confirmed by Western blot (**Figure 2C**).

To further examine the role of ETV4 in PD-L1 expression, we next established an ETV4 knockout cell line using CRISPR-Cas9 technology (**Figure 2D**). Genetic disruption of ETV4 significantly reduced mRNA levels of PD-L1 and PD-L2 in SUM-159 cells (**Figure 2E**). The decreased expression of PD-L1 after genetically silencing ETV4 was also confirmed by Western blot (**Figure 2F**). Consistently, the surface expressions of PD-L1 and PD-L2 were significantly reduced in ETV4 knockout cells as examined by flow cytometry (**Figure 2G and 2H**). Taken together, these data suggested that ETV4 was essential for PD-L1L2-SE-mediated PD-L1/L2 expression in cancer cells.

### ERK is required for ETV4 expression and PD-L1L2-SE activation

**Figure 3:**
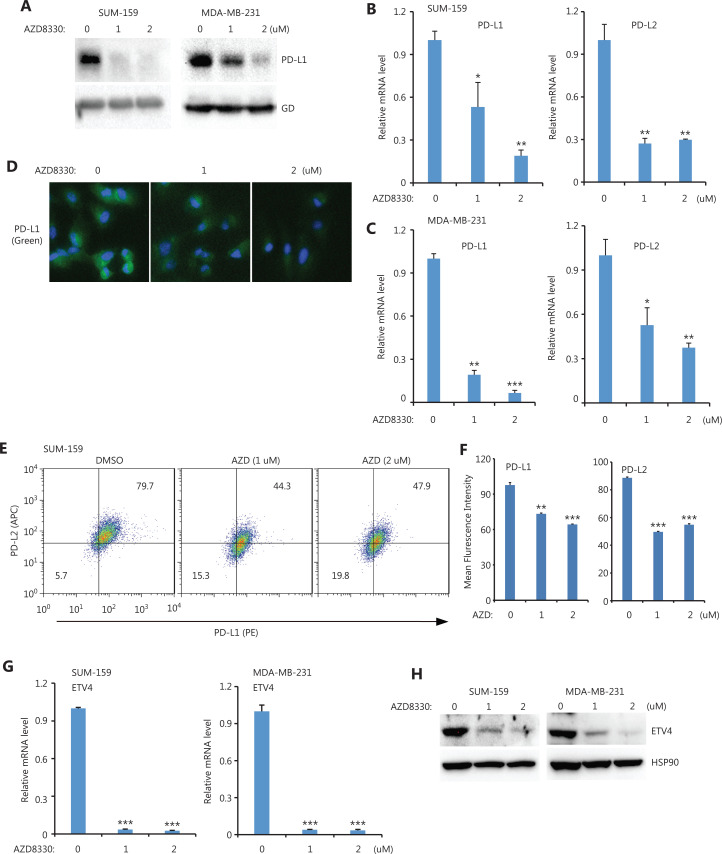
ERK inhibition downregulated PD-L1, PD-L2, and ETV4. (A) SUM-159 and MDA-MB-231 cells were treated with the ERK inhibitor, AZD8330, for 48 h and PD-L1 protein levels were measured by Western blot. Glyceraldehyde 3-phosphate dehydrogenase was used as the loading control. (B) PD-L1 and PD-L2 mRNA levels were examined by qRT-PCR in SUM-159 cells treated with AZD8330. (C) MDA-MB-231 cells were treated with AZD8330 for 48 h, and PD-L1 and PD-L2 mRNA levels were examined by qRT-PCR. (D) SUM-159 cells were treated with AZD8330 for 48 h at the indicated doses. PD-L1 expression (green) was examined by immunofluorescence. (E) MDA-MB-231 cells were treated with AZD8330 for 48 h at the indicated doses. The surface expression of PD-L1 (PE) and PD-L2 (APC) were examined by flow cytometry. (F) Summary of the mean fluorescence intensities from E. (G) SUM-159 and MDA-MB-231 cells were treated with AZD8330 at different doses for 48 h. ETV4 mRNA levels were determined by qRT-PCR. (H) SUM-159 and MDA-MB-231 cells were treated with AZD8330 for 48 h. ETV4 protein levels were measured by Western blot. HSP90 was used as the loading control. *P < 0.05; **P < 0.01; ***P < 0.001.

Because ETV4 is a TF, we next investigated which upstream pathways activated ETV4 and subsequently activated PD-L1/L2 expression. Four inhibitors (targeting the ERK, P38, JNK, and NFκB pathways) were used to examine the possible roles of these pathways in regulating PD-L1 and PD-L2 expressions. Notably, inhibition of ERK (AZD8330) but not P38 (SB203580), JNK (SP600125), or NFκB (PS-1145) greatly diminished PD-L1 protein levels in both SUM-159 and MDA-MB-231 cells (**Figure 3A and Supplementary Figure S1A**). As expected, the mRNA levels of PD-L1 and PD-L2 were also significantly decreased after treatment with the ERK inhibitor but not with the p38, JNK, or NFκB inhibitors in both SUM-159 and MDA-MB-231 cells (**Figure 3B, 3C, and Supplementary Figure S1B, S1C**). ERK inhibition profoundly reduced the surface expression of PD-L1 and PD-L2 in both SUM-159 and MDA-MB-231 cells, as assessed by immunofluorescence and flow cytometry (**Figure 3D, 3E, 3F, and Supplementary Figure S2**). Due to the role of ETV4 in SE-mediated PD-L1 and PD-L2 expressions, we next examined ETV4 expression after ERK inhibition. As shown in **Figure 3G**, ERK inhibition significantly reduced the mRNA expressions of ETV4 in both SUM-159 and MDA-MB-231 cells. Consistently, ETV4 protein levels were also greatly reduced by ERK inhibition in both SUM-159 and MDA-MB-231 cells (**Figure 3H**). Together, these results showed that ERK was required for PD-L1/L2 expression and ETV4 activation.

### BRAF and TAK1 are upstream kinases of the ERK/ETV4/PD-L1 (PD-L2) pathway

**Figure 4:**
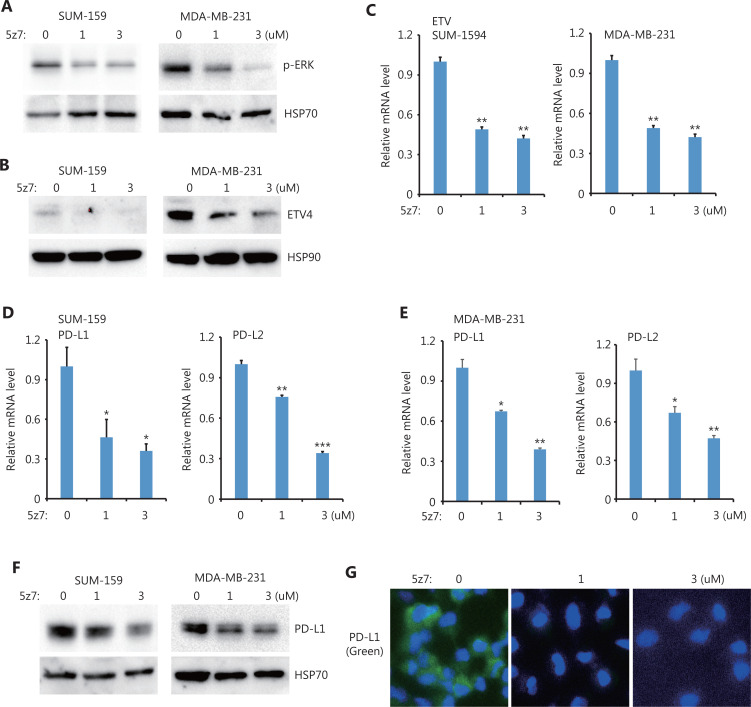
TAK1 inhibition downregulated ETV4, ERK phosphorylation, PD-L1, and PD-L2. (A) SUM-159 and MDA-MB-231 cells were treated with the TAK1 inhibitor, 5z7, for 48 h, and ERK phosphorylation was measured by Western blot. HSP70 was used as the loading control. (B) SUM-159 and MDA-MB-231 cells were treated with 5z7 for 48 h. ETV4 protein levels were measured by Western blot. HSP90 was used as the loading control. (C) SUM-159 and MDA-MB-231 cells were treated with 5z7 at different doses for 48 h. The qRT-PCR was used to measure ETV4 expression in each group. (D) SUM-159 cells were treated with 5z7 for 48 h. The qRT-PCR was used to determine PD-L1 and PD-L2 mRNA levels in each group. (E) MDA-MB-231 cells were treated with 5z7 for 48 h. The qRT-PCR was used to determine PD-L1 and PD-L2 expressions in each group. (F) SUM-159 and MDA-MB-231 cells were treated with 5z7 for 48 h. PD-L1 protein levels were measured by Western blot. HSP70 was used as the loading control. (G) Representative immunofluorescence images of PD-L1 expression (green) with and without 5z7 treatment. *P < 0.05; **P < 0.01; ***P < 0.001.

Next, we sought to determine the upstream kinases required for ERK activation and ERK-mediated PD-L1/L2 expression. Using small molecules to inhibit 3 potential kinases, TAK1 [(5z)-7-Oxozeaenol], BRAF (belvarafenib), and AKT (MK2206), we found that inhibiting TAK1 greatly reduced ERK activations in both SUM-159 and MDA-MB-231 cells (**Figure 4A**). In addition, ETV4 protein levels were also reduced by inhibiting TAK1 (**Figure 4B**), and ETV4 mRNA levels were also significantly decreased after TAK1 inhibition (**Figure 4C**). These results suggested that the reduced ETV4 protein level upon 5z7 treatment was due to decreased ETV4 transcription. As expected, inhibiting TAK1 significantly reduced PD-L1 and PD-L2 mRNA levels in both SUM-159 and MDA-MB-231 cells (**Figure 4D and 4E**). Western blot analysis showed that PD-L1 protein levels were dramatically decreased after TAK1 inhibition in both SUM-159 and MDA-MB-231 cells (**Figure 4F**). Immunofluorescence also showed greatly reduced PD-L1 expression in SUM-159 cells (**Figure 4G**). These results strongly indicated that TAK1 was an upstream regulator of ERK activation, ETV4 transcription, and PD-L1 expression.

**Figure 5:**
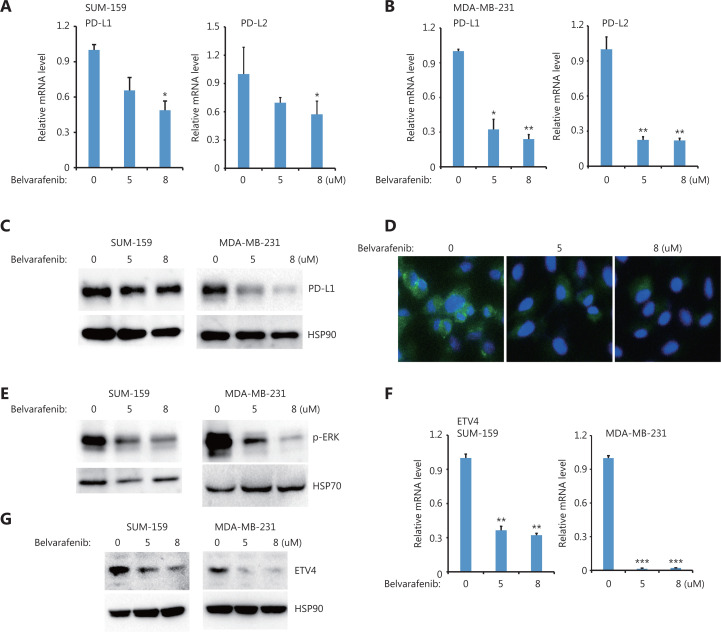
BRAF inhibition downregulated ETV4, and phosphorylated ERK, PD-L1, and PD-L2. (A) SUM-159 cells were treated with the pan-Raf inhibitor, belvarafenib, for 48 h. The qRT-PCR showed the expressions of PD-L1 and PD-L2 in each group. (B) MDA-MB-231 cells were treated with belvarafenib for 48 h. The qRT-PCR showed the expressions of PD-L1 and PD-L2 in each group. (C) SUM-159 and MDA-MB-231 cells were treated with belvarafenib for 48 h. PD-L1 protein levels were measured by Western blot. HSP90 was used as the loading control. (D) Representative immunofluorescence images of PD-L1 expression (green) with and without belvarafenib treatment. (E) SUM-159 and MDA-MB-231 cells were treated with belvarafenib for 48 h, and phosphorylated ERK levels were measured by Western blot. HSP70 was used as the loading control. (F) SUM-159 and MDA-MB-231 cells were treated with belvarafenib at different doses for 48 h. The qRT-PCR was used to determine ETV4 expression in each group. (G) SUM-159 and MDA-MB-231 cells were treated with belvarafenib for 48 h. ETV4 protein levels were measured by Western blot. HSP90 was used as the loading control. *P < 0.05; **P < 0.01; ***P < 0.001.

Moreover, we also found that inhibiting BRAF but not AKT significantly reduced PD-L1 and PD-L2 expressions in both SUM-159 and MDA-MB-231 cells (**Figure 5A, 5B and Supplementary Figure S3A**). PD-L1 protein levels were greatly reduced after inhibiting BRAF but not AKT in both SUM-159 and MDA-MB-231 cells (**Figure 5C and Supplementary Figure S3**), which is consistent with the immunofluorescence results (**Figure 5D**). BRAF inhibition also blocked ERK activation (**Figure 5E**), suggesting that ERK was downstream of BRAF. Moreover, BRAF inhibition significantly reduced ETV4 mRNA expression in both SUM-159 and MDA-MB-231 cells (**Figure 5F**). The reduced ETV4 mRNA levels following BRAF inhibition is consistent with reduced ETV4 protein levels after BRAF inhibition (**Figure 5G**). Together, these results showed that BRAF was required for ERK activation as well as for ETV4 and PD-L1 expressions, and provided compelling support for the critically important role of TAK1 and BRAF in ERK activation, ETV4 transcription, and PD-L1 expression.

### The extracellular signal that induces ERK activation and PD-L1/L2 expression is from αvβ3 integrin

**Figure 6:**
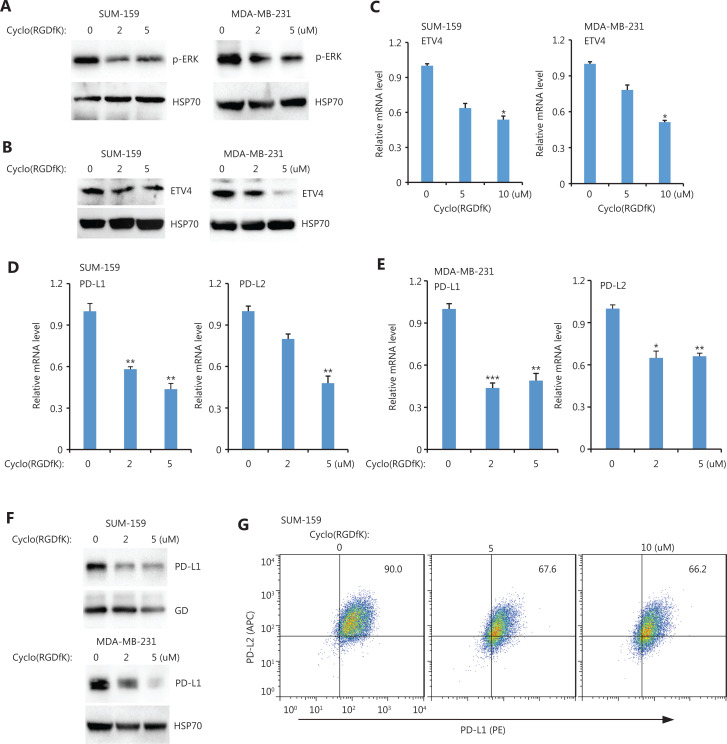
Inhibiting integrin αvβ3 downregulated ETV4, and phosphorylated ERK, PD-L1, and PD-L2. (A) SUM-159 and MDA-MB-231 cells were treated with the integrin αvβ3 inhibitor, cyclo(RGDfK), for 48 h. Phosphorylated ERK levels were measured by Western blot. HSP70 was used as the loading control. (B) SUM-159 and MDA-MB-231 cells were treated with cyclo(RGDfK) for 48 h. ETV4 protein levels were measured by Western blot. HSP90 was used as the loading control. (C) SUM-159 and MDA-MB-231 cells were treated with cyclo(RGDfK) for 48 h. The qRT-PCR showed ETV4 expression in each group. (D) SUM-159 cells were treated with cyclo(RGDfK) for 48 h. The qRT-PCR showed PD-L1 and PD-L2 expressions in each group. (E) MDA-MB-231 cells were treated with cyclo(RGDfK) for 48 h. The qRT-PCR showed PD-L1 and PD-L2 expressions in each group. (F) SUM-159 and MDA-MB-231 cells were treated with cyclo(RGDfK) for 48 h. PD-L1 protein levels were measured by Western blot. Glyceraldehyde 3-phosphate dehydrogase and HSP70 were used as loading controls for SUM159 and MDA-MB-231 cells, respectively. (G) SUM-159 cells were treated with cyclo(RGDfK) for 48 h at the indicated doses. The surface expressions of PD-L1 (PE-tagged) and PD-L2 (APC-tagged) were examined by fluorescence-activated cell sorting. *P < 0.05; **P < 0.01; ***P < 0.001.

The requirement of BRAF and TAK for ETV4 activation and subsequent PD-L1 expression suggested the presence of activating extracellular signals. We therefore examined 3 important extracellular signals including TGFβ (LY2109761), uPA (UK122), and integrin (Cyclo-RGDfK). Inhibiting TGFβ or uPA had minimal effects on PD-L1 expression (**Supplementary Figure S4**). However, blocking integrin signaling *via* Cyclo (RGDfK) dramatically reduced ERK activation in both SUM-159 and MDA-MB-231 cells (**Figure 6A**). Cyclo (RGDfK) treatment also greatly reduced ETV4 protein levels in both cell lines (**Figure 6B**). This reduction in ETV4 protein expression is consistent with the reduced ETV4 mRNA levels found after inhibiting integrin signaling (**Figure 6C**). Inhibiting integrin significantly diminished PD-L1/L2 expressions in both SUM-159 and MDA-MB-231 cells (**Figure 6D and 6E**). Western blot analysis showed PD-L1 protein levels were decreased after blocking integrin in both cell types (**Figure 6F**). Flow cytometry further confirmed reduced surface expression of PD-L1 and PD-L2 after inhibiting integrin signaling (**Figure 6G**). Taken together, these findings suggested a critical role for integrins in activating ERK and downstream ETV4-mediated PD-L1/L2 expression.

### Targeting the αvβ3 integrin/ERK/ETV4/PD-L1 (PD-L2) pathway blocks cancer cell immune evasion

**Figure 7:**
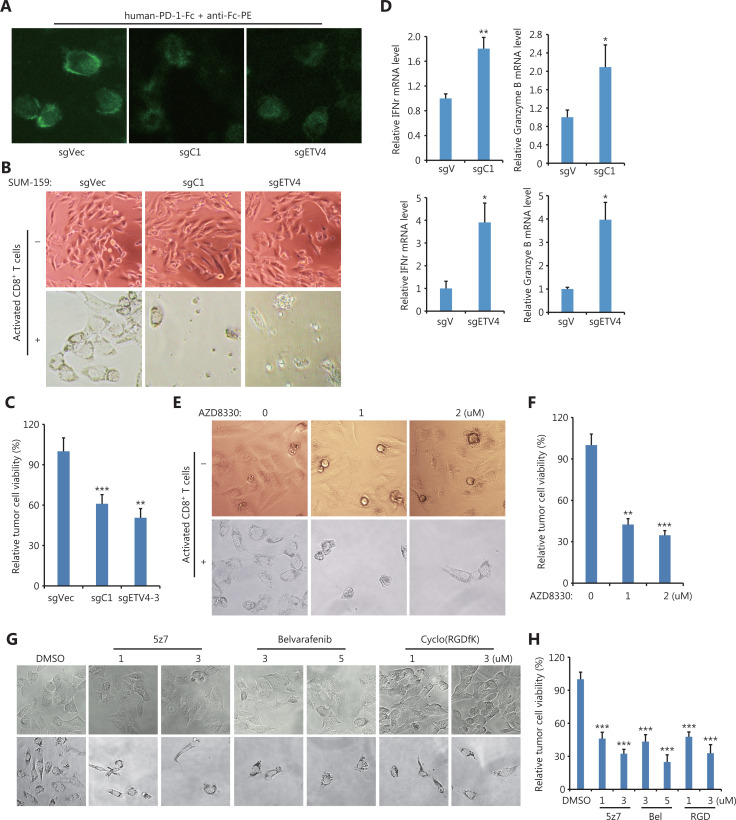
T cell-mediated cancer cell killing was enhanced after disrupting the PD-L1L2-SE-related pathway. (A) Representative images of PD-1 and PD-L1 interactions. Indicated sgC1, sgETV4, and sgVector cells were fixed and incubated with recombinant human PD-1-Fc protein for 2 h. The PE-conjugated anti-human Fc antibodies were incubated for 0.5 h. (B) Representative images of genetically modified SUM-159 cells after *in vitro* T cell-mediated killing assays. Cells transfected with sgC1, sgETV4, and sgVector were co-cultured with activated T cells for 48 h. T cells were washed away, and the remaining cancer cells were imaged. (C) Cells transfected with sgC1, sgETV4, and sgVector were co-cultured with activated T cells at a 1:5 ratio for 48 h. After washing away T cells, the remaining cancer cells were measured by the CCK-8 assay. (D) Cells transfected with sgC1, sgETV4, and sgVector were co-cultured with activated T cells for 48 h. T cells were collected, and the mRNA levels of IFNγ and granzyme B were examined by RT-PCR. (E) Representative images of SUM-159 cells treated with or without AZD8330 after T cell-mediated killing assays. SUM-159 cells were treated with AZD8330 for 24 h. After washing away AZD8330, the treated cells were co-cultured with activated T cells for 48 h. T cells were washed away, and the remaining cancer cells were imaged. (F) SUM-159 cells were treated with AZD8330 for 24 h. After washing away AZD8330, the treated cells were co-cultured with activated T cells for 48 h. T cells were washed away, and the remaining cancer cells were measured using the CCK-8 assay. (G) Representative images of SUM-159 cells treated with or without 5z7, Belvarafenib, and cyclo-(RGDfK) after T cell-mediated killing assays. SUM-159 cells were treated with inhibitors for 24 h. After washing away inhibitors, the treated cells were co-cultured with activated T cells for 48 h. T cells were washed away, and the remaining cancer cells were imaged. (H) SUM-159 cells were treated with inhibitors for 24 h. After washing away inhibitors, the treated cells were co-cultured with activated T cells for 48 h. T cells were washed away, and the remaining cancer cells were measured using the CCK-8 assay. *P < 0.05; **P < 0.01; ***P < 0.001.

Although immune checkpoint blockade (ICB) targeting PD-L1/PD-1 with antibodies has achieved impressive clinical success, ICB strategies still need to be improved. First, we measured the interaction between PD-1 and PD-L1 proteins by using recombinant human PD-1-Fc protein as well as anti-human Fc antibodies-conjugated with PE. **Figure 7A** shows that fluorescence images clearly demonstrated the engagement of PD-1 and PD-L1 in sgC1 and sgETV4 cells was greatly reduced, when compared to that in control sgVec cells (**Figure 7A**). Next, we tested whether targeting the identified pathway that drives SE-mediated PD-L1 expression could enhance antitumor immune reactions, by performing *in vitro* T cell-mediated killing assays. **Figure 7B** shows a significant portion of control cells (sgVec) survived during co-culture with activated T cells, while genetic knockout of the C1 element (sgC1) or ETV4 (sgETV4) made the cells sensitive to T cell-mediated killing (**Figure 7B and 7C**). Consistent with the role of PD-L1, T cells co-cultured with sgC1 or sgETV4 cells were more activated than T cells co-cultured with sgVec cells, as shown by increased IFNγ and granzyme B production (**Figure 7D**). Additionally, SUM-159 cells were sensitive to T cell-mediated killing after being treated with the ERK inhibitor, AZD8330, indicating the critical role of ERK signaling in PD-L1 expression (**Figure 7E and 7F**). Inhibiting TAK1, BRAF, or integrin also rendered SUM-159 cells sensitive to T cell-mediated killing (**Figure 7G and 7H**). These results further supported the conclusion that the integrin/TAK1/BRAF/ERK/ETV4 signaling axis was involved in SE-mediated PD-L1 expression. Thus, targeting this pathway could achieve immune cell-mediated cancer cell death. These results therefore suggested that the identified pathway could be a potential target for ICB.

## Discussion

ICB targeting PD-L1/PD-1 has been used in a broad spectrum of cancers to renormalize and reset antitumor immunity^21^. Although its overall therapeutic effect is promising and some patients show long-term remission, ICB faces several major challenges such as low response and adverse effects^22^. Antibody-based ICB can effectively block PD-L1 on the cell surface, but intracellular PD-L1 likely cannot be targeted. These issues should be seriously considered, as several recent reports have demonstrated a nuclear function of PD-L1^23–25^. In addition, disrupting PD-L1 on both cancer and normal cells also blocks the beneficial functions of PD-L1 and leads to side effects. Thus, it is ideal to target a unique pathway through which cancer cells, but not normal cells, induce PD-L1 expression. Previously, we found that PD-L1L2-SE was specifically activated in cancer cells, where it was required for PD-L1 expression^16^. Here, we further mapped the core region of PD-L1L2-SE and showed that the TF ETV4 was important for PD-L1 expression (**Figure 1 and Figure 2**). Furthermore, ETV4 and PD-L1 were activated by the extracellular matrix (ECM) through the integrin/BRAF/TAK1/ERK pathway (**Figures 3–6**). Disrupting this pathway by either genetic knockouts or inhibitors made cancer cells sensitive to T cell-mediated killing (**Figure 7**). These findings indicated that PD-L1L2-SE itself as well as its related pathways might be potential pharmacological targets to specifically disrupt PD-L1 in cancer cells.

The ECM and its resident cells such as endothelial, mesenchymal, and immune cells comprise the tumor microenvironment^26^. It is now recognized that the tumor microenvironment plays a pivotal role in tumor growth and metastasis, and also determines responses to antitumor therapies including ICB^27^. In many malignancies, the ECM from tumor cells and cancer-associated fibroblasts compromise up to 60% of the tumor mass^28^. Various ECM molecules such as fibrillar collagens, fibronectin, elastin, hyaluronan, and laminins are frequently observed in solid tumors^29^. Cancer cell-matrix adhesion is mediated by interactions between membrane-associated integrins and the arginine-glycine-aspartic (RGD) motif displayed on many ECM proteins. In the present study, we found that Cyclo (RGDfK), which is a potent inhibitor of αvβ3 integrin, significantly diminished PD-L1 and ETV4 expressions as well as immune evasion (**Figure 6**). This is consistent with a recent report that showed αvβ3-integrin regulated PD-L1 expression and was involved in immune evasion^30^. Although αvβ3-integrin is required for SE-mediated PD-L1/L2 expression, it might be insufficient to activate PD-L1L2-SE because αvβ3-integrin activation is widely observed in cultured cell lines, while PD-L1L2-SE-induced PD-L1/L2 expression is less frequently observed. Therefore, besides αvβ3-integrin, other factors or downstream pathways might collaboratively induce PD-L1L2-SE.

In our investigation of pathways downstream of αvβ3-integrin, we unexpectedly found that both TAK1 and BRAF were required for ETV4 activation and PD-L1 expression (**Figure 3 and Figure 4**). MEK inhibition in combination with anti-PD-L1 promotes T cell infiltration and anti-tumor activity, however the effect of MEK inhibition on PD-L1 expression is inconsistent^31,32^. Here, we found that ERK activation was required for ETV4 expression and PD-L1L2-SE activation (**Figure 5**). This may partially explain the synergic effects between MEK inhibitors and anti-PD-L1 antibodies. TAK1 and BRAF are both mitogen-activated protein kinase kinases (MAP3Ks). Inhibition of TAK1 or BRAF could efficiently block ERK activation and downstream PD-L1 expression. It is interesting that cancer cells need both MAP3Ks to activate SE-mediated PD-L1 expression. However, further studies need to address the distinct roles of TAK1 and BRAF in PD-L1L2-SE activation.

By analyzing the TFs that bind to a core region of PD-L1L2-SE, we found a potentially important TF (ETV4) for PD-L1L2-SE-mediated PD-L1 expression. Molecular studies have confirmed the role of ETV4 in PD-L1 expression (**Figure 2**). ETV4 is a TF that is known to be involved in tumor development and progression^20^. Our results suggested an important role for ETV4 in immune evasion by activating PD-L1. Although we observed higher ETV4 protein levels in SUM159 and MDA-MB-231 cells compared with MCF7 cells, MCF7 cells expressed ETV4 but not PD-L1. Thus, it was possible that other TFs cooperatively worked with ETV4 to activate PD-L1L2-SE.

## Conclusions

We identified a core DNA region of approximately 3 kb within PD-L1L2-SE that was essential for PD-L1 expression and immune evasion. We further found that ETV4 was an essential TF for PD-L1L2-SE-mediated PD-L1 expression. In breast cancer, ETV4 was transcriptionally activated by the ECM *via* the integrin/BRAF/TAK1/ERK pathway. In solid tumors, the ECM delivered immune evasion signals to cancer cells *via* integrin, which activated MAP3Ks (TAK1 and BRAF) to phosphorylate ERK. Phosphorylated-ERK entered the nucleus and induced transcription of ETV4. ETV4 opened PD-L1L2-SE, inducing PD-L1 and PD-L2 expression. PD-L1/L2-expressing cancer cells inhibited T cell function and evaded T cell-mediated killing (**Supplementary Figure S5**). Our findings suggested a cancer-specific pathway that activated a specific SE. Importantly, PD-L1L2-SE-associated pathways might be potential targets for disrupting PD-L1 expression and immune evasion.
