# Supplementary material for: Super-enhancer receives signals from the extracellular matrix to induce PD-L1-mediated immune evasion via integrin/BRAF/TAK1/ERK/ETV4 signaling
Source: Cancer Biol Med. 2021 Oct 9;19(5):669–84. doi: 10.20892/j.issn.2095-3941.2021.0137 (PMC9196059; doi:10.20892/j.issn.2095-3941.2021.0137)
Supplement: Supplementary file 1 [file cbm-19-669-s001.pdf]

Supplementary materials

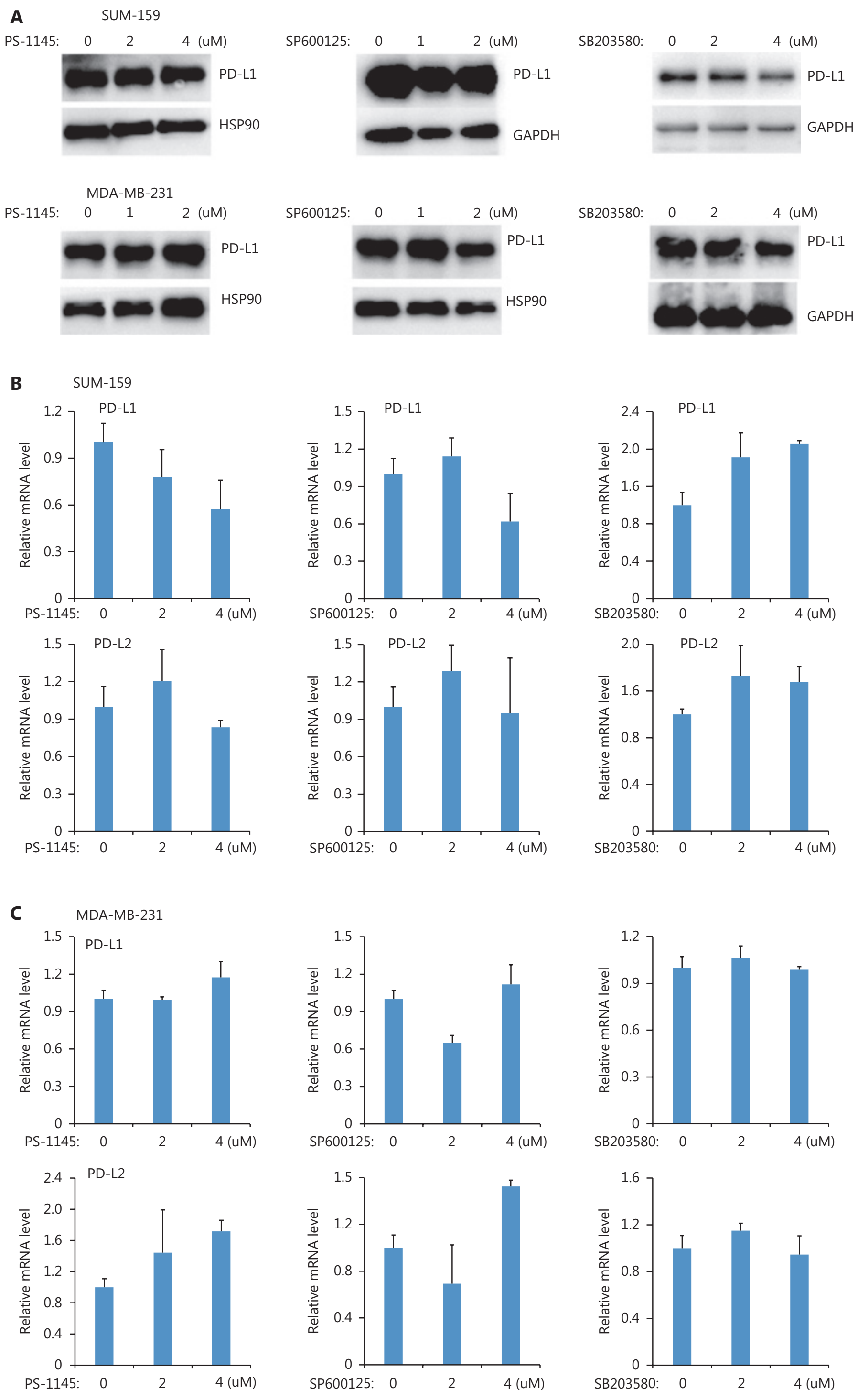

Figure S1 The effect of P38, JNK, and IKK inhibition on PD-L1 expression. (A) SUM-159 and MDA-MB-231 cells were treated with PS-1145, SP600125, and SB203580 for 48 h. PD-L1 and PD-L2 levels were examined in each group by Western blot. (B) SUM-159 cells were treated with PS-1145, SP600125, and SB203580 for 48 h. The qRT-PCR showed PD-L1 and PD-L2 mRNA expressions in each group. (C) MDA-MB-231 cells were treated with PS-1145, SP600125, and SB203580 for 48 h. The qRT-PCR showed PD-L1 and PD-L2 mRNA expressions in each group.

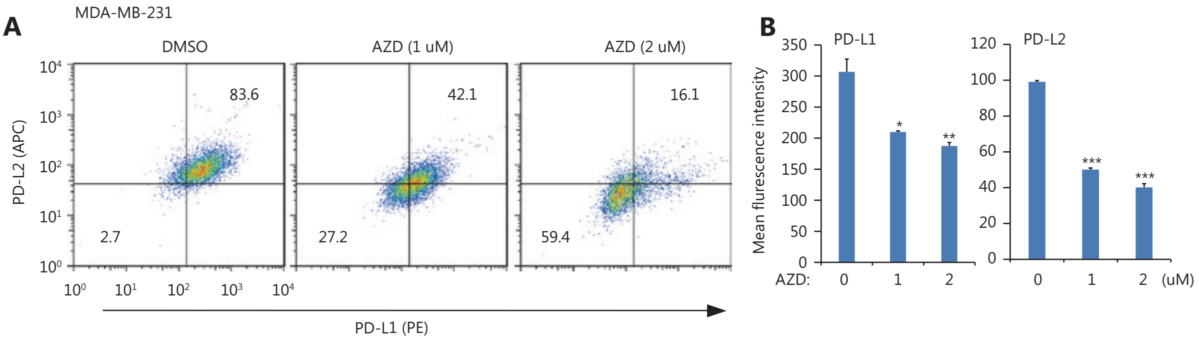

Figure S2 The effect of ERK inhibition on PD-L1 expression. (A, B) MDA-MB-231 cells were treated with AZD for 48 h at the indicated doses. Flow cytometry was used to examine PD-L1 and PD-L2 surface expressions in each group. *P < 0.05; **P < 0.01; ***P < 0.001.

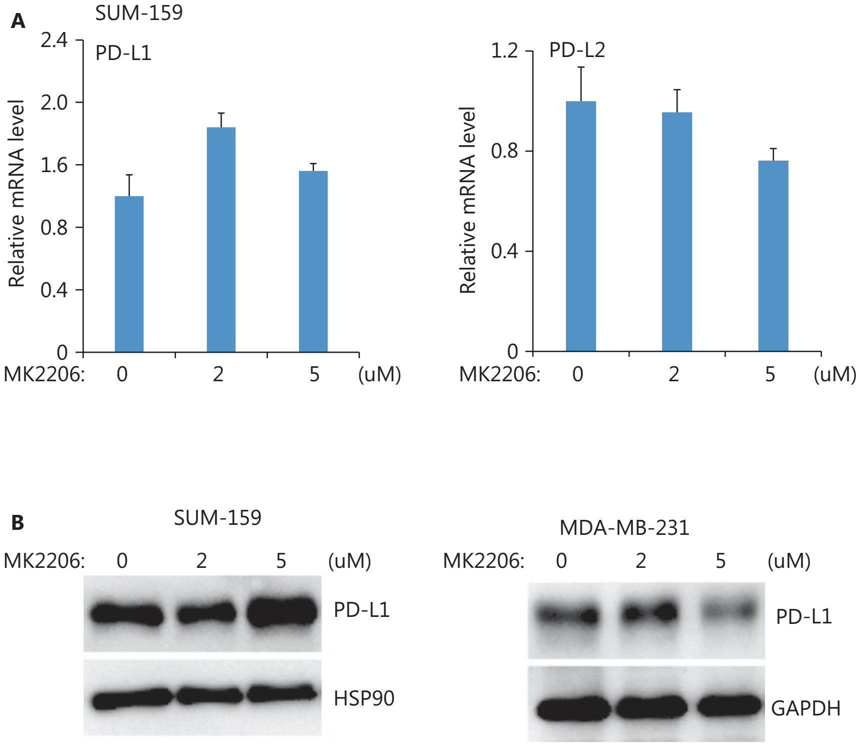

Figure S3 The effect of AKT inhibition on PD-L1 expression. (A) SUM-159 cells were treated with MK2206 for 48 h. The qRT-PCR showed PD-L1 and PD-L2 mRNA expressions in each group. (B) SUM-159 cells were treated with MK2206 for 48 h. Western blots were used to show PD-L1 and PD-L2 protein expressions in each group.

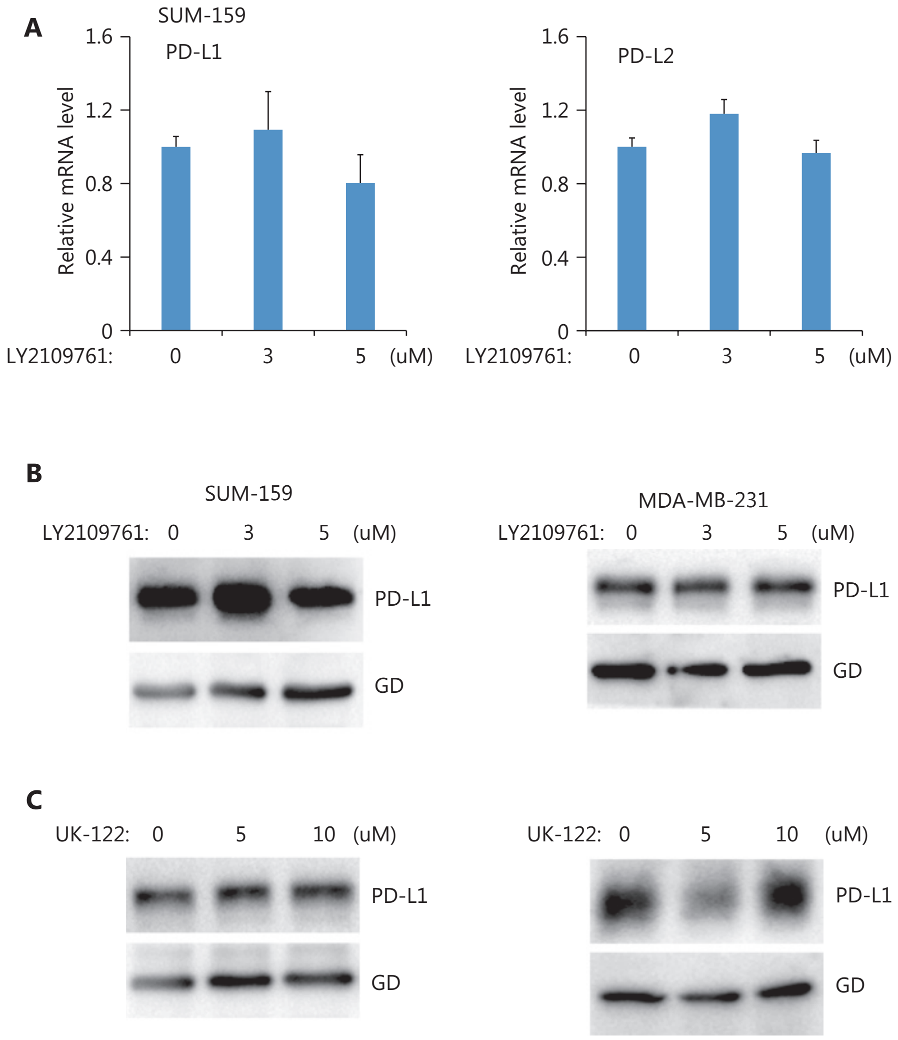

Figure S4 The effect of TGFβ and uPA inhibition on PD-L1 expression. (A) SUM-159 cells were treated with LY2109761 for 48 h. The qRT-PCR showed PD-L1 and PD-L2 mRNA expressions in each group. (B) SUM-159 cells were treated with LY2109761 for 48 h. Western blots were used to show PD-L1 protein expressions in each group. (C) SUM-159 cells were treated with UK-122 for 48 h. Western blots were used to show PD-L1 protein expression in each group.

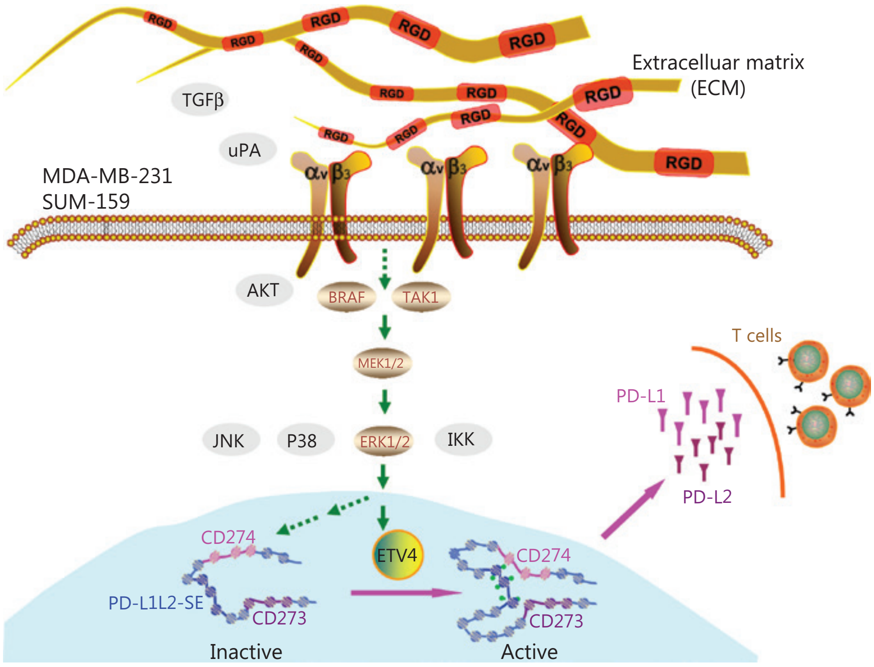

Figure S5 A working model showing how the extracellular matrix induces PD-L1 and PD-L2 expressions *via* PD-L1L2-SE. In the mass of a solid tumor, the extracellular matrix delivers immune evasion signals to cancer cells *via* integrins. Upon recognition of the RGD motif by αvβ3-integrin, integrin activates MAP3Ks such as TAK1 and BRAF to further phosphorylate ERK. Phosphorylated-ERK translocates into the nucleus where it induces ETV4 transcription. ETV4 can open the super-enhancer PD-L1L2-SE, inducing PD-L1 and PD-L2 expressions. When cancer cells present PD-L1 and PD-L2 on their surface, they inhibit T cell function and evade T cell-mediated killing.
